# Molecular Epidemiology of Staphylococcus aureus in China Reveals the Key Gene Features Involved in Epidemic Transmission and Adaptive Evolution

**DOI:** 10.1128/spectrum.01564-22

**Published:** 2022-10-03

**Authors:** Zhen Xu, Chao Yuan

**Affiliations:** a Department of Toxicology and Sanitary Chemistry, School of Public Health, Tianjin Medical University, Tianjin, PR China; b Tianjin Key Laboratory of Environment, Nutrition and Public Health, Tianjin Medical University, Tianjin, PR China; c Center for International Collaborative Research on Environment, Nutrition and Public Health, School of Public Health, Tianjin Medical University, Tianjin, PR China; University of Minnesota

**Keywords:** *Staphylococcus aureus*, molecular epidemiology, pangenome, core genome, adaptive evolution

## Abstract

Staphylococcus aureus is a Gram-positive pathogen that causes various infections in humans and domestic animals. In China, S. aureus is the most common Gram-positive pathogen that causes clinical infections. However, there are few comprehensive genome-based molecular epidemiology studies to investigate the genotypic background of the major S. aureus clones that are epidemic in China. Here, four S. aureus isolates that were recovered from hospital personnel were sequenced. In combination with whole-genome sequencing (WGS) data of 328 S. aureus strains as references, we performed a comprehensive molecular epidemiology study to reveal the molecular epidemic characterization of S. aureus that is epidemic in China. It was found that 332 S. aureus isolates were phylogenetically categorized into 4 major epidemic groups with different epidemiology phenotypes. Each group has exclusive features in virulence genotypic profiles, antimicrobial resistance genotypic profiles, core and pangenome features representing the differences involved in genetic features, evolutionary processes, and potential future evolutionary directions. Moreover, a comparative core genome analysis of 332 S. aureus isolates indicated several key genes that contributed to differences in molecular epidemic characterization and promoted the adaptive evolutionary process of each group. This study provides a comprehensive understanding of molecular epidemiological characteristics and adaptive evolutionary directions of major S. aureus clones that are epidemic in China.

**IMPORTANCE** Staphylococcus aureus is an important Gram-positive pathogen that is epidemic worldwide and causes various infections in humans and domestic animals. However, there has been relatively little research on comprehensive molecular epidemiology in China. In this research, we reconstructed the phylogenetic relationship based on whole-genome data of strains almost all over China, screened for resistance and virulence genes, and took core and pan genome analysis to perform a comprehensive molecular epidemiology study of S. aureus that is epidemic in China. Our results highlight that there are 4 major epidemic groups with different epidemiology phenotypes after phylogenetic categorization with exclusive genetic features in virulence genotypic profiles, antimicrobial-resistance genotypic profiles, and core and pangenome features, and we found key gene features involved in epidemic transmission and adaptive evolution. Our findings are critical in describing molecular characteristic profiles of S. aureus infection, which could update existing preventive measures and take appropriate strategies.

## INTRODUCTION

Staphylococcus aureus is one of the most important Gram-positive pathogens of humans and domestic animals. As a commensal, S. aureus is often present asymptomatically on groups of the human body, such as skin, skin glands, and mucous membranes, including the nostrils and pharynx of healthy individuals (1–3). Because of its ability to spread worldwide and cause outbreaks in both hospitals and the community (4, 5), S. aureus has become one of the most intensively studied microorganisms in the world (6–8), emphasizing the need for more in-depth analysis and research. In China, S. aureus is the most common Gram-positive pathogen that causes clinical infections ranging from minor skin infections to life-threatening diseases (9); however, there has been relatively little research on comprehensive molecular epidemiology in China.

S. aureus was first identified and characterized in 1880, with extremely high mortality of invasive infections until the introduction of penicillin at the beginning of the 1940s (10, 11). Methicillin-resistant isolates of S. aureus (MRSA) have emerged and are epidemic worldwide. Three groups of MRSA have been described, including hospital-associated MRSA (HA-MRSA), community-associated MRSA (CA-MRSA), and livestock-associated MRSA (LA-MRSA) (12, 13). MRSA ST5-II and ST239-III are the dominant hospital-associated genotypes worldwide, and ST8-IV (USA300) and ST5-IV are the main community-associated genotypes (12). In addition, MRSA ST59-IV/V is the predominant community-associated clone in Asia. ST398 and ST9 are the major genotypes of livestock-associated MRSA (6, 8).

The prevalence and epidemiology of S. aureus are continually changing, with novel clones appearing in different geographical regions (3, 5, 8, 14). Genome plasticity promotes the rapid evolution of S. aureus to accommodate variations in the environment by modulating virulence, antimicrobial resistance, and environmental adaption (12, 15, 16). However, there is no genome-based molecular epidemiology study to investigate the genotypic background of the major S. aureus clones that are epidemic in China. Therefore, understating the molecular epidemiology of S. aureus is critical in describing molecular characteristics profiles of S. aureus infection, which could update existing preventive measures and assist in implementing appropriate strategies.

In this study, 4 MRSA isolates were recovered from hospital personnel in Tianjin, China, and sequenced. Combined with whole-genome sequencing (WGS) data of 328 S. aureus isolates that were mainly isolated in China (92.7%), we reconstructed the phylogenetic relationship of 332 S. aureus strains, screened for resistance and virulence genes, and performed core and pangenome analyses, revealing the molecular epidemic characteristics of S. aureus that is epidemic in China.

## RESULTS AND DISCUSSION

### Phylogenetic analysis revealed the epidemic profiles of S. aureus in China.

**FIG 1:**
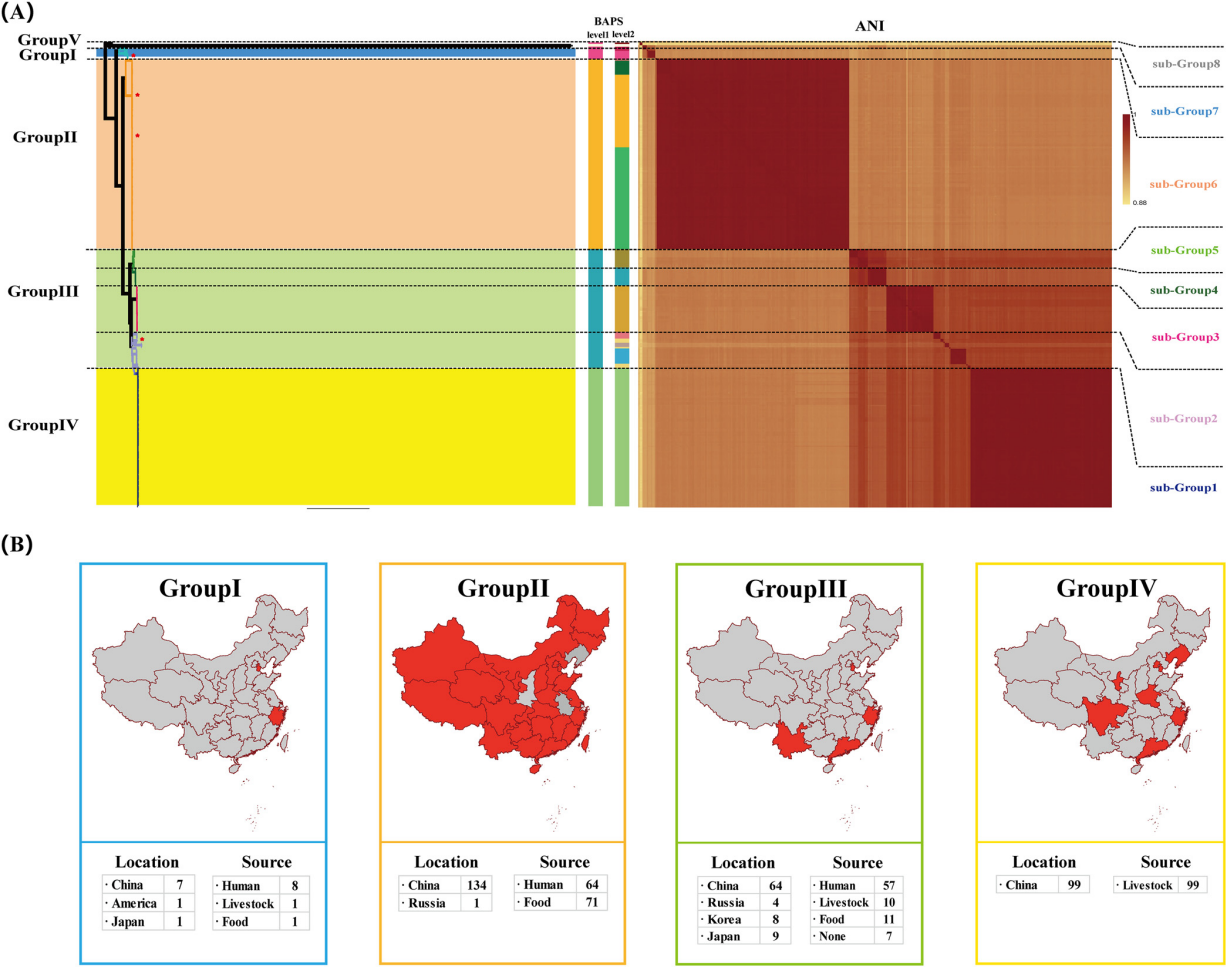
Phylogenetic relationship and epidemic information of 332 Staphylococcus aureus strains. (A) ML phylogeny was constructed based on SNPs across 854 single-copy core gene families shared by the 332 Staphylococcus aureus genomes. The heatmap of STs, clonal complex, and BAPS is shown on the right of the chart. The color of each subgroup corresponds to the branch color on the tree. (B) The epidemic information of 332 Staphylococcus aureus strains. Isolation sites (province) of S. aureus are labeled on the map of China with dark red.

In this research, 4 S. aureus strains were recovered from hospital personnel in Tianjin (municipalities in China) and sequenced. Combining this data with the reference data of 328 published S. aureus genomes (China, *n* = 304; Asian cities, *n* = 23; United States of America, *n* = 1) from the NCBI database, phylogenetic analysis was performed (detailed information of the 332 S. aureus isolates is shown in Table S1 in the supplemental material). To assess the phylogenetic relationship of S. aureus, a maximum-likelihood phylogenetic tree was reconstructed based on 854 concatenated single-copy core gene families from 332 S. aureus genomes (Fig. 1A). Genomic similarities among strains were explored by genetic population structure analysis using Bayesian analysis of population structure (BAPS) in two levels, and the average nucleotide identity (ANI) value (Fig. 1A) was calculated to estimate the genetic distance between strains at the genomic level. Three-hundred and thirty-two S. aureus isolates were divided into 5 BAPS classes (level 1) or 16 BAPS classes (level 2), which were consistent with the results of the ANI value. Detailed information about sequence type (ST) and clonal complex (CC) was also shown and statistics can be found in Fig. S1 in the supplemental material. Combined with the epidemiological information and the structure of the phylogenetic tree, we divided 332 S. aureus isolates into 5 groups (group I to V) or 8 subgroups (subgroup 1 to 8) (Fig. 1A). The correlation between the two classification methods was as follows (labeled in Fig. 1A): group I (subgroup 7), group II (subgroup 6), group III (subgroups 2, 3, 4, and 5), group IV (subgroup 1). S. aureus in group V is not discussed in this study due to a limited number of isolates and the absence of epidemiological information.

The phylogenetic analysis determined three major epidemic lineages of S. aureus in China. Four S. aureus strains isolate from our study were widely distributed in these three lineages (labeled in Fig. 1A). Thus, it is suggested that hospital personnel might be significantly affected by epidemic clones of S. aureus in China. The majority (78%, *n* = 7) of S. aureus isolates in group I were human clinical isolates that were recovered from China, and our MRSA ST398-V-t034 was located in this clade. MRSA ST398 was reported to be the predominant livestock-associated clone in the United States, Canada, Europe, and Asia (13); however, in our study, most S. aureus isolates of group I were recovered from clinic and hospital personnel in China. It is alarming to find that LA-MRSA clones are widely epidemic among clinic and hospital personnel in China. Except for one isolate from Russia, 134 out of 135 (99%) S. aureus isolates of group II (subgroup 6) were isolated from China. A total of 64 of 135 isolates were clinical isolates, and 71 were recovered from food. Huh and Chung reported that MRSA ST59 IV/V were the predominant community-associated clones in Asia (14). Consistent with their study, MRSA ST59 IV/V was China’s dominant clone. Our MRSA ST59-II-t437 and ST59-IV-t437 were located in this clade.

S. aureus isolates belonging to group III (subgroups 2, 3, 4, and 5) showed diverse STs and BAPS classes, and clones of this clade exhibited varied sources (Fig. 1B). Therefore, S. aureus isolates belonging to group III revealed the potential capacity to accommodate different countries and environments to cause an epidemic. Our MRSA ST15-V-t084 isolate was located in subgroup 2 of group III, and many foreign isolates recovered from other Asian countries were in subgroup 2. Based on our results, the MRSA ST15-V-t084 isolate could be regarded as an epidemic clone of Asia, and subgroup 2 might represent a transmission process of MRSA clones between China and other Asian countries. In particular, S. aureus isolates belonging to group IV were isolated from livestock in China. S. aureus isolates belonging to groups III (subgroups 2, 3, 4, and 5) and IV (subgroup 1) were phylogenetically closely related and formed a large clade, which suggested the host evolutionary adaptation process of S. aureus between humans and livestock. Epidemiological information of four groups is shown in Fig. 1B and is listed in Table S1.

### Pan and core genome analyses revealed the genetic characteristics among S. aureus in China.

**FIG 2:**
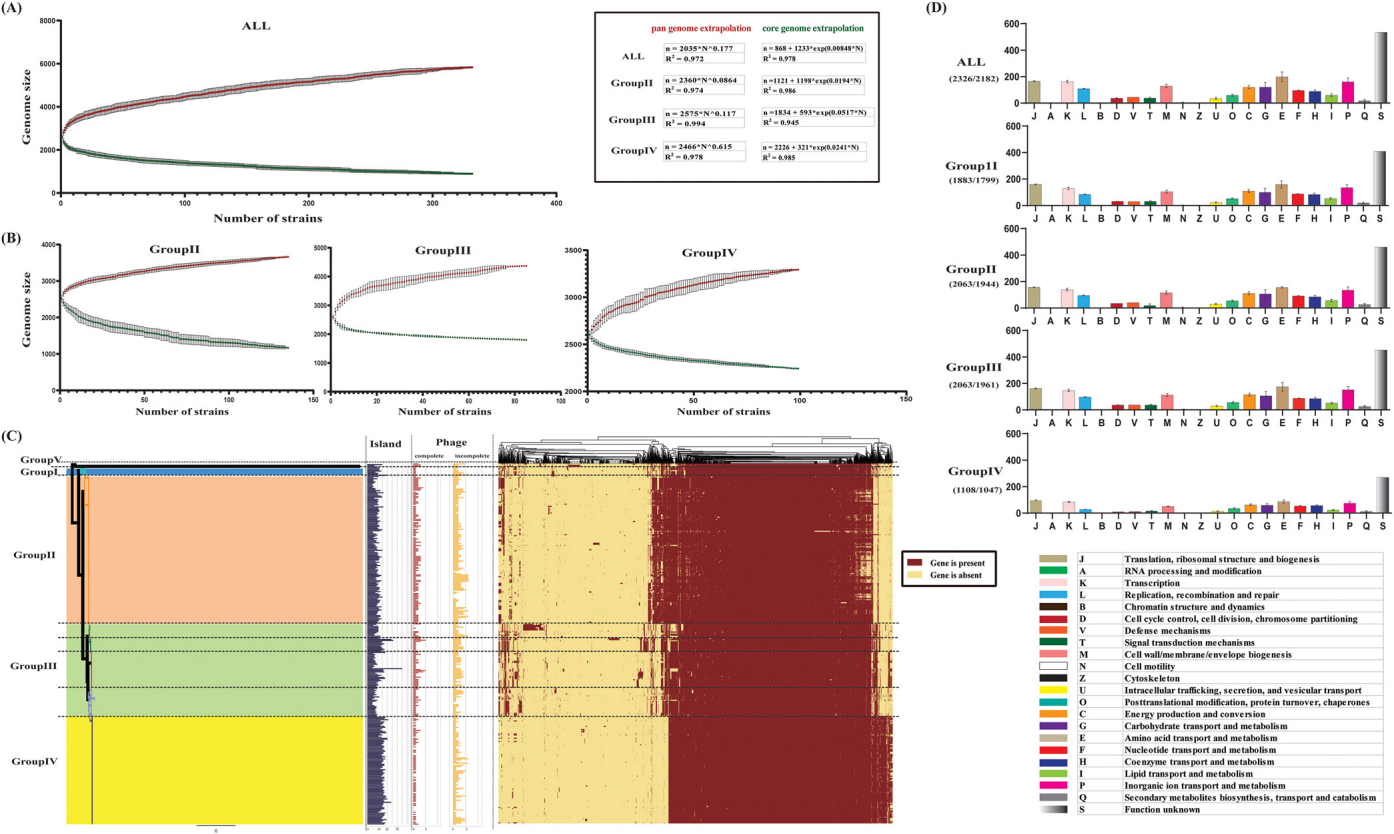
Core and pangenome analysis of 332 Staphylococcus aureus strains. Core and pangenome curves of all strains (A) and different groups (B) show the different downward trends of the core gene families and the upward trend of the pan-gene families with the increased number of genomes. The error bars indicate the standard deviation of the number of core and pan-gene families. The fitting mathematical functions of the core and pangenome curves are shown in a black frame. (C) Cluster map of the pan genome of S. aureus. Island and phage of S. aureus are also shown. (D) COG categories of all strains and different groups for each gene family set.

Pan and core genome analyses were performed to analyze the molecular epidemic characteristics of 332 S. aureus strains. The 332 genomes contained 5,832 gene families classified into three classes as follows: core genes, accessory genes, and unique genes. The core genome was formed by 890 gene families (15.3%) shared by all strains, while the remaining 4,942 gene families (84.7%) formed the variable genome. The variable genome of 332 isolates of S. aureus was constituted by the unique genome (1,327 gene families that were specific to a single strain) and the accessory genome (3,615 gene families present in more than one strain). The cluster map of the pangenome all-blast-all divided 332 S. aureus strains into 4 groups (Fig. 2C), which were consistent with our phylogenetic classification of 332 strains. A large number of uncharacterized unique genes in the pangenome of S. aureus warranted further attention.

We illustrated the core and pangenome of all S. aureus strains to further analyze the core and pangenome gene features (Fig. 2A). Each group was analyzed based on Heap’s law pangenome model (Fig. 2B), and a fitted curve was drawn (due to the limited number of isolates, *n* < 10, group I is not discussed). Heap’s law pangenome model (*n* = κN^γ^) was used to analyze the pan genome of 332 S. aureus isolates, and the increased power law (γ = 0.177) was determined. The pangenome analysis showed a clear linear upward trend, representing an open pangenome species. The pangenome of groups II, III, and IV also showed a clear linear upward trend; however, a different power law suggested that each group may have a different open degree level. As shown in Fig. 2B, the pangenome of group IV had a much higher power law (γ = 0.615); however, the power law dropped dramatically in group II (γ = 0.0864). Thus, strains of group IV contained a large source gene pool and had the potential to adapt to new niches by acquiring novel genetic elements. Moreover, the genetic diversity of strains in group II gradually decreased during epidemics, potentially becoming a closed genome in future evolution. In addition, results of pangenome all-blast-all are shown in Fig. 2C (detailed information about pan-blast-screen results is shown in Table S4 in the supplemental material) combined with the results of mobile genetic elements (MGEs) detection (islands and phages), and discernible differences among the four groups were observed. The results suggested that group I, group II, and groups III and IV were the three major lineages with different epidemiological characteristics and evolved independently under different genetic backgrounds in China.

Conversely, we found significant differences in the downtrend of core genome curves of each group of S. aureus isolates, which suggested a potential association between core genome differences and epidemiological variation. Functional enrichment of the core genome of each group based on clusters of orthologous group (COG) analysis was performed (Fig. 2D). To investigate the differences in core genome between different groups of S. aureus isolates, we also took COG analysis on a core gene pool of 4 groups. Results showed that the core genome could be categorized into 22 gene families after COG annotation. Compared with all 332 genomes in the core gene pool, similar gene annotation ratios and gene number distributions were detected in different categories of each group. However, “T: signal transduction mechanisms” (Fisher’s exact test, *P* = 0.045) of the core genome of group II had a significantly decreased enrichment degree, and “L: replication, recombination and repair” (Fisher’s exact test, *P* = 0.001) of the core genome of group IV had a notably reduced enrichment degree. These results revealed that there were indeed differences between the core genome of each group of S. aureus isolates in China; however, the gene number differences did not have statistical significance after COG functional enrichment analysis. Therefore, we hypothesized that these differences might exist in specific cellular pathways of bacteria.

### Virulence genotypic profiles revealed the pathogenicity of S. aureus in China.

**FIG 3:**
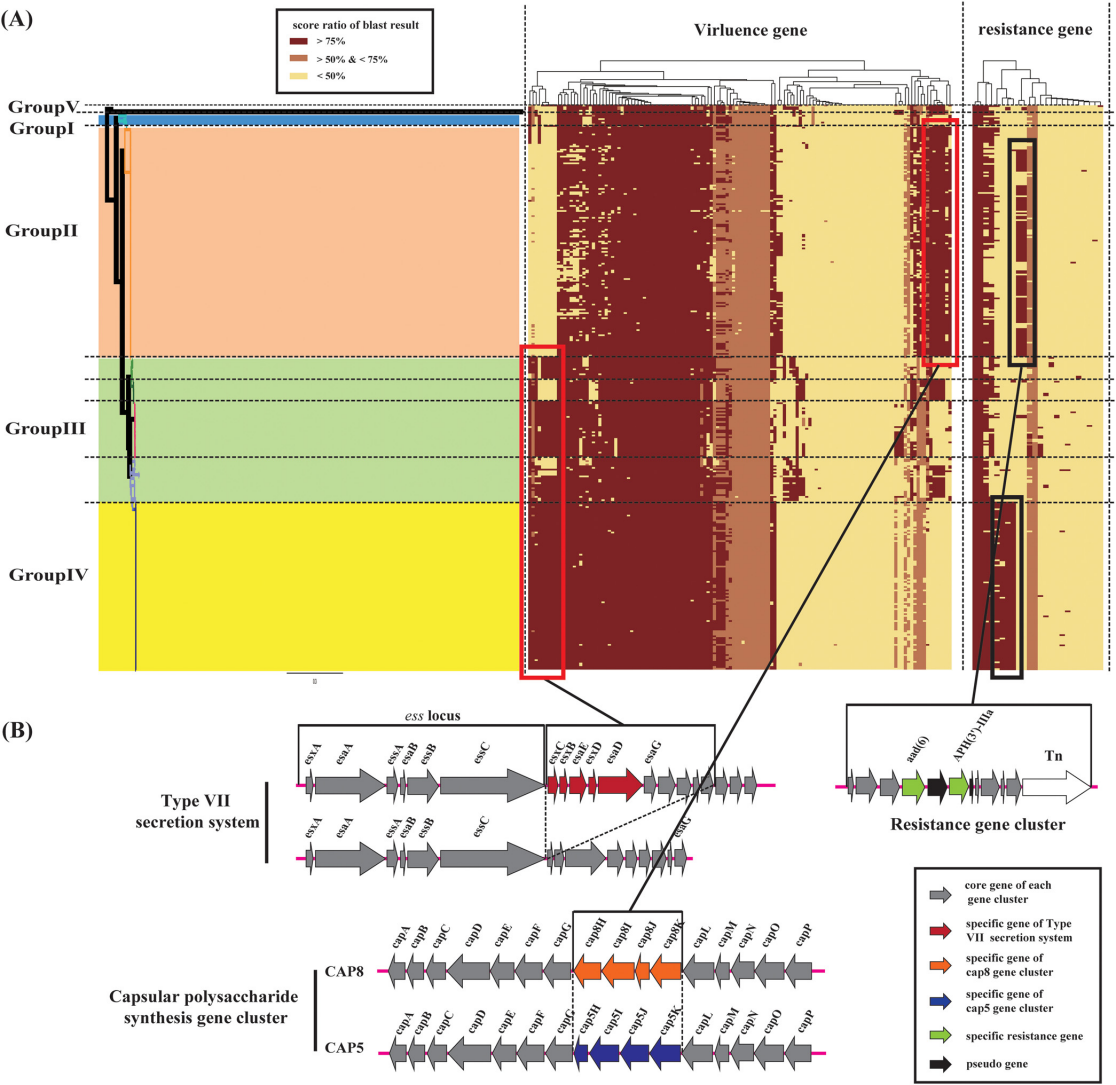
The virulence and resistance genotypic profiles of 332 Staphylococcus aureus strains. (A) Heatmap of screen result and color coding for each gene corresponding to the score ratio of blast result of each genome. (B) The genetic structure of specially distributed virulence gene clusters and resistance gene clusters.

Virulence genes of S. aureus were investigated to identify key pathogenic characteristics of isolates in different groups. All 332 S. aureus genomes were locally aligned against the Virulence Factors Database (VFDB) and cluster map as shown in Fig. 3A. The results revealed two apparent differences among S. aureus strains (shown in the red frame). Virulence genes (VFG049739, VFG049753, VFG049793, VFG049727, VFG049714, VFG049701, VFG002410, VFG002411, and VFG001799) were mainly distributed in strains of group III and group IV, including the *map* gene and a group of type VII secretion system (T7SS) gene clusters. The group of T7SS consisted of *esxB*, *esxC*, *esxD*, *esaE*, *esaD*, and several *esxG* genes (Fig. 3B), which mediate persistent abscess lesions and assistant-bacterial competition (17, 18). The majority of isolates of group III and all strains of group IV had complete T7SS gene clusters, whereas S. aureus isolates belonging to groups I and II had incomplete T7SS gene clusters (*ess* locus) (Fig. 3A). This finding was consistent with the previous study (19) and revealed the different pathogenicity and adaptivity of isolates between groups III and IV and other groups.

Conversely, virulence genes (VFG001324, VFG049820, VFG001802, VFG001304, VFG001305, VFG001306, and VFG001307), which were known as *isdG*, *chp*, *seb*, and specific genes of type 8 capsular polysaccharide gene cluster (*cap8H*, *cap8I*, *cap8G*, *cap8K*), were mainly found in S. aureus isolates belonging to group II. Capsular polysaccharide (CP) has been reported as an essential virulence gene of S. aureus. More than 80% of the clinical isolates produce type 5 CP (CP5) or type 8 CP (CP8) (20). CP5 and CP8 encode vital extracellular structures, which conferred S. aureus isolates the ability to evade host immunity (20, 21). Similarly, our results revealed that the CP8 gene cluster was mainly identified in group II and III (subgroups 2 and 4), whereas the CP5 gene cluster was distributed in group I and IV (Fig. 3B). Varied virulence genes found in S. aureus isolates belonging to groups II and IV indicated the different capabilities to evade host immunity; meanwhile, various virulence genes identified in group II revealed the pathogenic diversity of S. aureus in group II. Detailed information from the Virulence Factors Database screen results is listed in Table S3 in the supplemental material.

### Resistance genotypic profiles revealed the diverse resistance of S. aureus in China.

Methicillin-resistant S. aureus (MRSA) has become a significant pathogen worldwide and is increasingly detected in hospitals and communities (7). Antimicrobial-resistant genes profiles of 332 S. aureus strains (Fig. 3A) were investigated, and the majority of isolates of group II specifically had the following two antimicrobial resistance genes: ARO:3002628 (*aad(6)*) and ARO:3002647 (*APH(3′)-IIIa*). *aad(6)* and *APH(3′)-IIIa* encode aminoglycoside-modifying enzymes (AMEs), which contribute to aminoglycoside resistance. Moreover, almost all strains of group IV exclusively had three resistance genes, ARO:3000250 (*ermC*), ARO:3002704 (*fexA*), and ARO:3000179 (*tetL*), which conferred erythromycin resistance, florfenicol resistance, and tetracycline resistance, respectively. Strains belonging to groups I and II harbored fewer antimicrobial resistance genes than those belonging to groups III and IV. Detailed information regarding CARD screen results is listed in Table S4.

**FIG 4:**
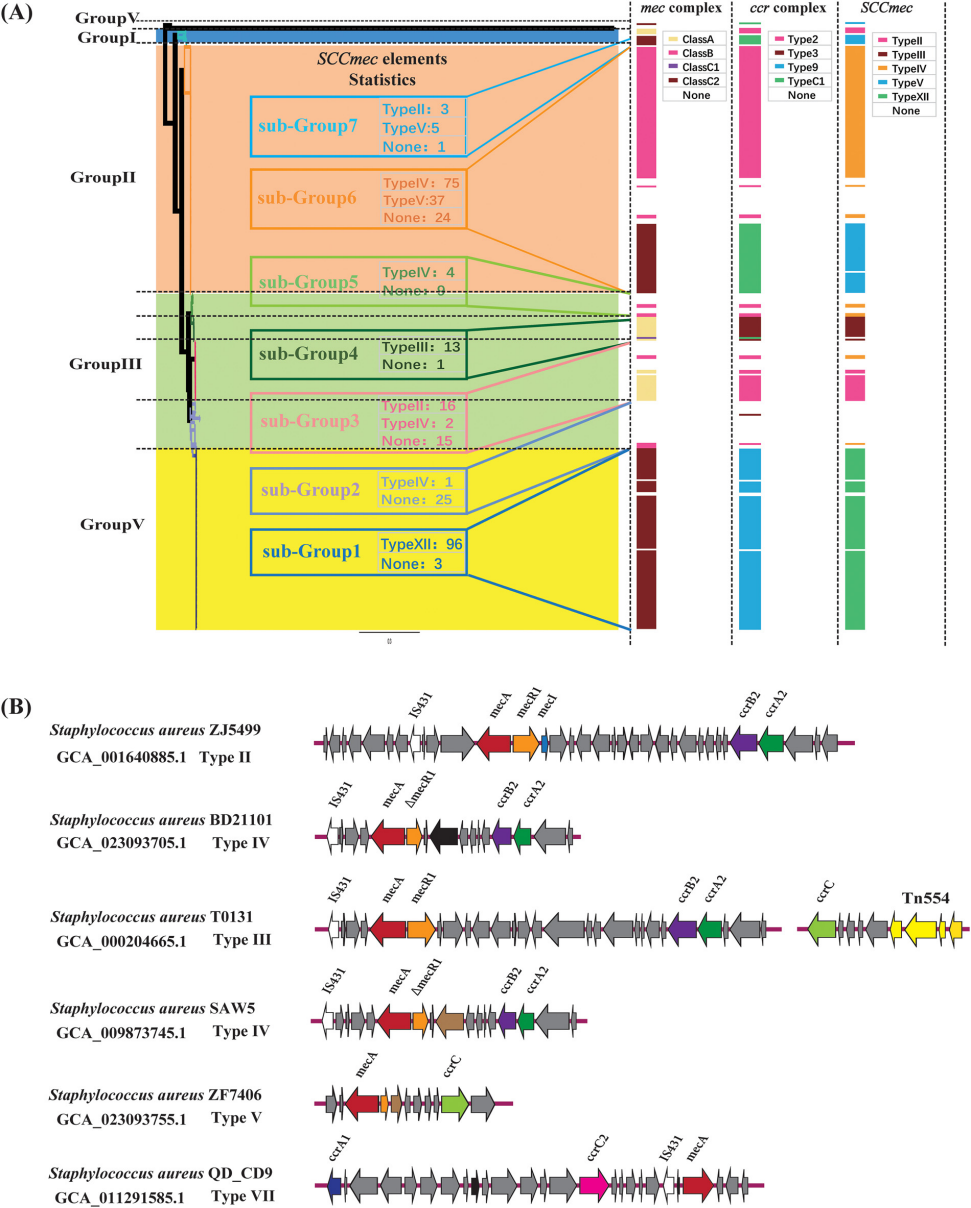
The genotypic profiles of SCC*mec* elements across all 332 Staphylococcus aureus strains. (A) Heatmap and statistics information of the 5 types of SCC*mec* elements among all 332 S. aureus strains. (B) The genetic structure of 5 types of SCC*mec* elements.

The *mecA* gene mediates the methicillin resistance of S. aureus and is located on a mobile genetic element named staphylococcal cassette chromosome *mec* (SCC*mec*). SCC*mec* types I, II, and III are hospital associated, and types IV and V are community-acquired SCC*mec* types. To date, SCC*mec* elements from I to XIV have been identified (22, 23). In this study, SCC*mec* elements were found in 82.4% (112/136) of S. aureus isolates in group II and 97.0% (96/99) of isolates in group IV. The carriage rate of SCC*mec* of group II and IV was much higher than the rate of group III (41.9%, 36/86). Moreover, subgroup 2, formed by Asian clones, showed the lowest carriage rate (3.8%, 1/26) (Fig. 4A). S. aureus isolates in groups II and IV were mainly recovered from China, which suggested the severe threat of methicillin-resistant S. aureus in China. Additionally, five types of SCC*mec* elements were identified in 332 S. aureus strains, including SCC*mec* types II, III, IV, V, and XII. It is worth noting that SCC*mec* type IV was widely identified in S. aureus isolates in four groups, and SCC*mec* types III and XII were exclusively found in group IV and subgroup 4. Therefore, SCC*mec* XII was the dominant SCC*mec* type identified in LA-MRSA in China, which is dramatically different from the SCC*mec* types found in LA-MRSA of other countries (13, 23). SCC*mec* types and antimicrobial-resistant genes were varied in the S. aureus isolates of each group, which might be associated with the genetic diversity of each group and different evolutionary processes. The genetic structure of 5 types of SCC*mec* elements among 332 S. aureus strains was shown in Fig. 4B.

### Comparative core genome analysis revealed the differences in genetic characteristics among S. aureus strains in China.

**FIG 5:**
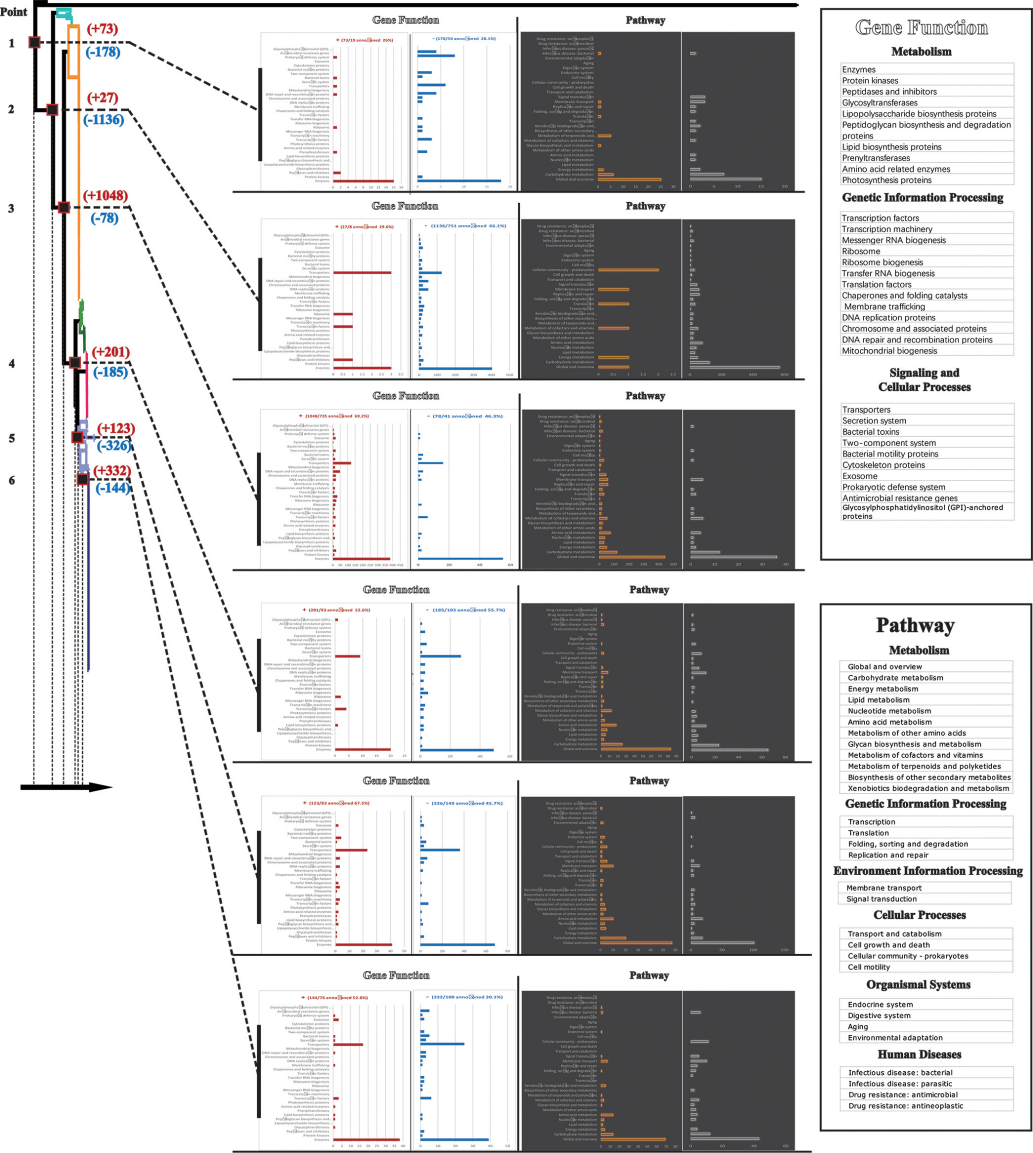
Comparative core genome analysis at branch point on the phylogenetic tree figured out the differences in genetic characteristics among each subgroup. Each branch point is colored with a dark red frame and numbered from point 1 to point 6 following the direction of the arrow. The number of core genome gene differences at each point is labeled, an increased number of genes is labeled with dark red, and a decreased number of genes is marked by blue. A bar chart shows KEGG annotation, and based on two classification methods, “gene function” and “pathway” (detailed information of the two classification methods is shown in the black frame on the right), an increased number of genes is shown in dark red and orange and a decreased number of genes is shown in blue and gray. KEGG annotation ratio is also shown in the figure.

The research above hypothesized that the core genome differences contributed to different epidemiological phenotypes. However, no gene number differences were found. Therefore, we believed that the differences might exist in specific cellular pathways. Comparative genomic analysis was performed on the core genome of each subgroup to find these specific pathways. We focused on differences in the core genome that arise at key branching points on the phylogenetic tree, and key branching points were generated between two adjacent subgroups. The core genome differences at key branching points could reveal the adaptive evolutionary processes of S. aureus isolates of 7 subgroups at the gene level (Fig. 5). The branch points were numbered in the direction of the arrows from points 1 to 6, which represented core genome differences between two adjacent subgroups. The decreased gene number at point 2 and increased gene number at point 3 were much higher than that at the other points, which indicated that subgroups 6, 7, and 8 were derived from three independently evolved lineages in China. In contrast, the increased and decreased numbers of core gene families at points 4, 5, and 6 were relatively low and stable, which suggested a relatively smooth evolutionary process in subgroups 1 to 5 (also known as groups III and IV).

Conversely, we took KEGG annotation analysis to investigate the cellular pathway in which core genome differences were involved. Results were presented by two methods: [gene function] and [pathway], and the annotation rate was shown (Fig. 5). At points 2 and 3, core genome differences were mainly involved in [gene function] (“transporters,” “enzymes,” “DNA repair and recombination proteins,” “DNA replication proteins”) and [pathway] (“signal transduction,” “membrane transport,” “replication and repair,” “metabolism of cofactors and vitamins,” “amino acid metabolism,” “carbohydrate metabolism”). At other points, core genome differences were mainly involved in [gene function] (“transporters,” “enzymes”) and [pathway] (“signal transduction,” “membrane transport,” “carbohydrate metabolism”). In addition, each point exclusively had some core genome difference genes, and the data was not shown. In general, core genome differences between subgroups were mainly involved in environmental signal response and key substance synthesis and metabolism, suggesting an adaptive evolutionary process under different genetic backgrounds.

**FIG 6:**
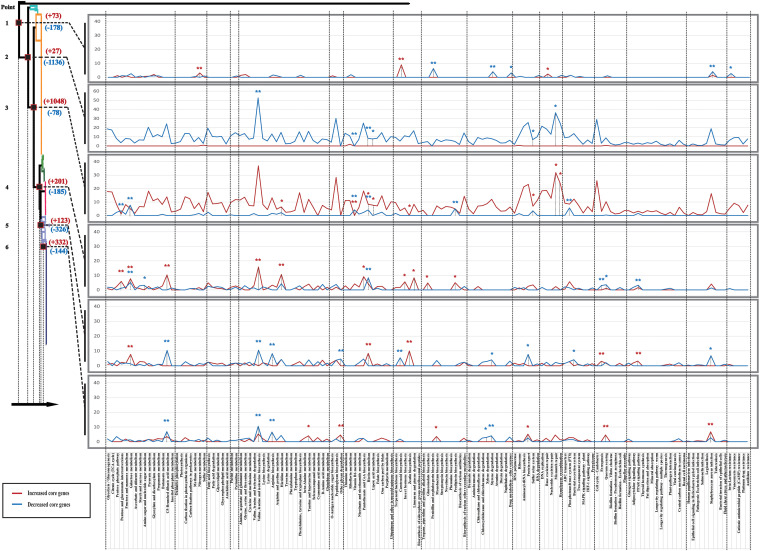
Screen results of KEGG annotation analysis at branch point on the phylogenetic tree. Each point is colored with a dark red frame and numbered from point 1 to point 6 following the direction of the arrow, and a corresponding line chart of RER values of each KEGG pathway is shown. Relative enrichment ratio (RER) is introduced to describe the correlation between gene enrichment and a pathway. The relative enrichment ratio formula is RER = (number of genes annotated in this pathway) × 100/(total number of genes in this pathway based on the KEGG database). High RER values represent an effective enrichment; an increased number of genes is shown in dark red, and a decreased number of genes is shown in blue (*, Fisher’s exact test *P* value < 0.05; **, Fisher’s exact test *P* value < 0.01. The color corresponds to the RER value on the line chart). Detailed information on the KEGG pathway is shown at the bottom.

**FIG 7:**
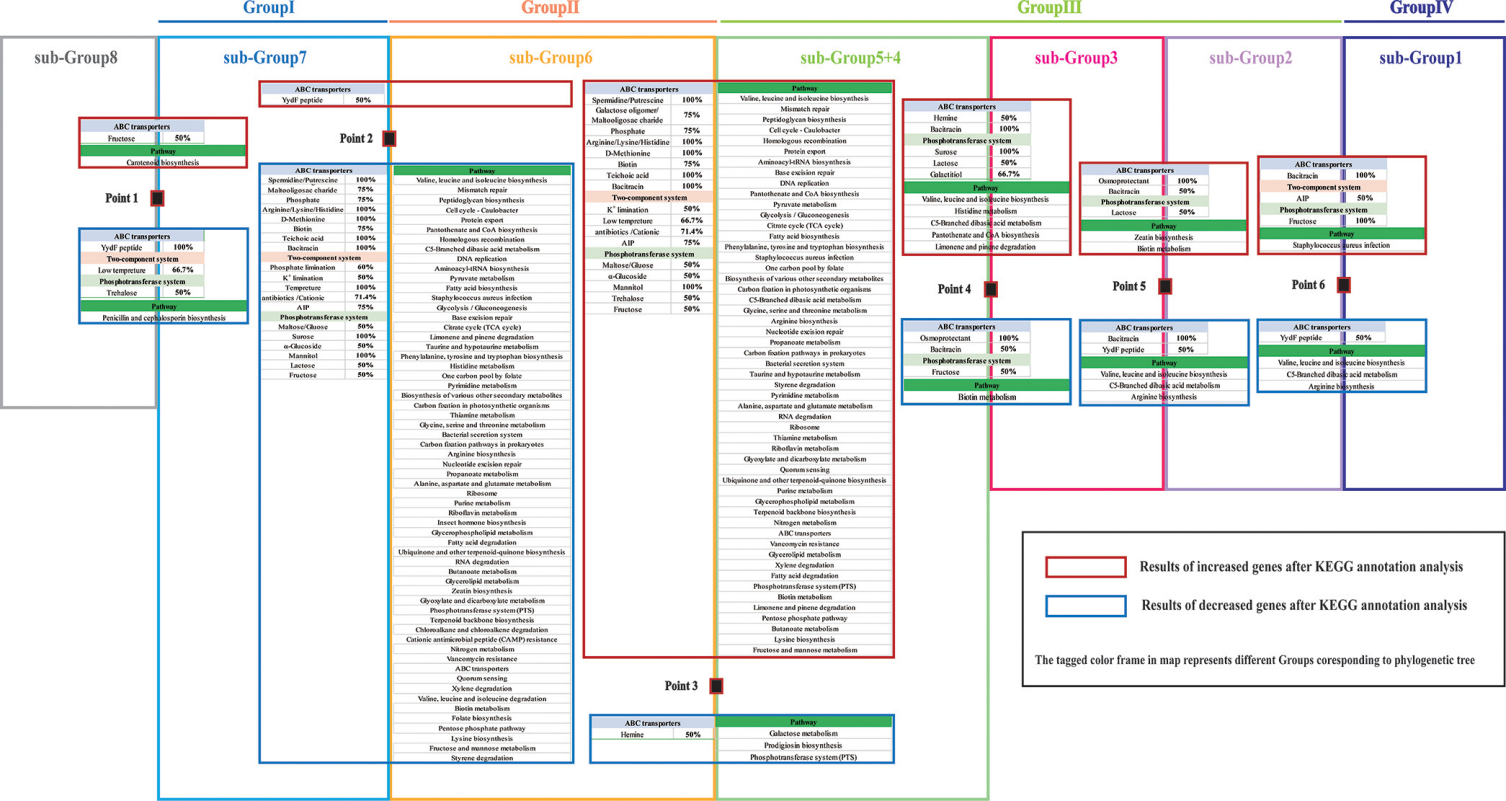
Results of comparative core genome analysis at branch point on the phylogenetic tree. With KEGG annotation analysis, increased and decreased genes are listed in dark red and blue frames, located above and below each point. Each group and each subgroup is colored as in Fig. 1.

To analyze the relationship between the adaptive evolutionary process under different genetic backgrounds and epidemiological characteristics of S. aureus in China at the gene level, we focused on specific KEGG pathways and genes that were associated with environmental signal response and key substance synthesis and metabolism. There were background differences in the number of genes involved in different pathways of bacteria. Thus, relative enrichment ratio (RER) was introduced to describe the correlation between a gene enrichment and a pathway. The formula was as follows: RER = (number of genes annotated in this pathway) × 100**/**(total number of genes in this pathway); high RER values represented an effective enrichment. We found that all differences in the core genome between two adjacent groups were enriched into 141 pathway classifications based on RER values. RER values of each pathway were calculated to normalize the results of the KEGG annotation analysis, and the graph was plotted at each point as shown in Fig. 6. In each graph, there are several distinct peaks corresponding to different pathways at each point, which represent an adequate enrichment in the corresponding pathway and suggests a high probability of phenotypic changes along this pathway; Fisher’s exact test was used to compare the pathways between groups. To take a more accurate analysis, we used RER = 8 as the criterion for screening, and the effective enrichment pathway of core genome differences at each point is listed in Fig. 7. Conversely, the “ABC transporters,” “two-component system,” and “Phosphotransferase system (PTS)” were three vital categories of genes that were involved in response to environmental signals of bacteria. Therefore, core genome differences involved in these three pathways at each point were investigated separately, and increased/decreased genes (≥50%) are shown in Fig. 7. At point 2 and point 3, many core genome differences were observed between subgroups 5 + 4, 6, and 7, and genes were mainly involved in the pathway and environmental signaling. The findings were consistent with the result of the core genome analysis mentioned above. In addition, the differences also indicated that strains in group I, group III, and group IV had great environmental and host adaptive ability, which was consistent with epidemiological results in Fig. 1B. Whereas, at points 4 to 6, relatively few core genome differences were observed, which suggests that strains of subgroup 1, subgroup 2, subgroup 3, and subgroup 4 + 5 may have diverged from a common ancestor. Conversely, combined with epidemiological data, core genome differences at points 4, 5, and 6 also revealed a process by which core gene families change during the evolutionary process from a vast host to a single host, including “ABC transporters: hemin, bacitracin, osmoprotectant, YydF peptide,” “Phosphotransferase system (PTS): fructose, lactose, sucrose, galactitol,” and “pathway: valine, leucine, and isoleucine biosynthesis, histidine metabolism, C5-branched dibasic acid metabolism, pantothenate and CoA biosynthesis, limonene and pinene degradation, biotin metabolism, S. aureus infection, arginine biosynthesis, and zeatin biosynthesis.” Moreover, the pathways contained genes involved in the adaptive evolutionary process of S. aureus and promoted the transmission between humans and livestock. Screen and statistic results of core genome differences based on KEGG annotation are shown in Table S5 and S6 in the supplemental material. Core genomes analysis of each subgroup after KEGG annotation are also show in Fig. S2 in the supplemental material. Screen and statistics results of the core genome of each subgroup based on KEGG annotation are shown in Table S7 in the supplemental material.

### Conclusions.

In this study, we performed a molecular epidemiological analysis on 332 S. aureus isolates to provide a comprehensive understanding of the molecular epidemiological characteristics of S. aureus in China from the genomic perspective. Three main lineages were established by phylogenetic analysis, including group I, group II, and groups III and IV. Different ST, CC, BAPS, and phenotypes were clarified in each group, representing the genotypic features of three major epidemic clones of S. aureus in China. Pangenome analysis showed that group II, group III, and group IV presented a clear linear upward trend. However, their power laws varied dramatically. Different power laws suggested different levels of genetic plasticity of each group and revealed the unique evolutionary direction of each group in the future. High levels of genetic plasticity were due to many MGEs, genome rearrangements, and horizontal genes, and the high genetic plasticity contributed to the expansion of gene pools. Accordingly, the epidemiological diversity of each group was guaranteed.

Besides, we described the virulence profiles among 332 S. aureus strains. Most strains of group III and all strains of group IV have a complete T7SS gene cluster, whereas the rest of the strains only had the *ess* locus of T7SS. Lost genes of T7SS were involved in persistent abscess lesions and interbacterial competition. In addition, the capsular eight-specific gene cluster (*cap8H*, *cap8I*, *cap8G*, and *cap8K*) was explicitly detected in S. aureus strains belonging to group II and some strains of group III (subgroup 2 and subgroup 4), which suggested the differences in host specificity and hosted immune escape ability of S. aureus in each group. In general, virulence gene differences conferred varied pathogenicity to S. aureus in each group in China. In addition, different antimicrobial resistance genotypic profiles were determined in each group. SCC*mec* II, III, IV, V, and XII were detected in S. aureus isolates in China. Specific SCC*mec* elements were exclusively identified in some groups; we also found that SCC*mec* type XII was the dominant SCC*mec* element associated with LA-MRSA in China. In general, a severe threat of MRSA was observed in China.

Finally, core genome differences at branching points mainly involved specific KEGG pathways and environmental signaling. The number of core genome differences at points 2 and 3 was significantly higher than that in other points, which indicated that three lineages separated by points 2 and 3 in the phylogenetic tree evolved independently in China. In contrast, core genome differences at points 4,5, and 6 were relatively low, which suggested a relatively slow evolutionary process (group III and group IV). Besides, core genome differences at points 4, 5, and 6 also revealed a process by which core gene family changes were evolved from a wide host to a single host, including “ABC transporters: hemin, bacitracin, osmoprotectant, Yydf peptide,” “phosphotransferase system (PTS): fructose, lactose, sucrose, galactitol,” and “pathway: valine, leucine and isoleucine biosynthesis, histidine metabolism, C5-branched dibasic acid metabolism, pantothenate and CoA biosynthesis, limonene and pinene degradation, biotin metabolism, S. aureus infection, arginine biosynthesis, and zeatin biosynthesis”.

In summary, 332 S. aureus isolates from China were phylogenetically categorized into four major epidemic groups, and our 4 S. aureus strains were epidemic in these 4 groups. Moreover, the genomic differences in each group were consistent with their epidemiological characterization based on KEGG annotation. In addition, genome analysis of 332 S. aureus isolates promoted our understanding of molecular epidemiological characteristics and adaptive evolutionary directions of major clones in China.

## MATERIALS AND METHODS

### Bacterial strains and DNA extraction.

Bacterial strains in this research were listed in Table S1 in the supplemental material. The 4 S. aureus isolates were recovered from hand and nasal swabs of hospital personnel in 2 hospitals in Tianjin, China. All strains were stored at −80°C in preservation medium supplemented with 50% (vol/vol) glycerol and cultured at 37°C in tryptone soy broth (TSB). Bacteria extraction kit (CWBIO Co., Ltd, China) for DNA extractions from each strain was used according to the manufacturer’s instructions.

### Genome sequencing and data processing.

The genomic sequencing was performed using Solexa paired-end sequencing technology (Illumina, Little Chesterford, Essex, UK), with a 90- to 100-fold coverage depth. The reads were *de novo* assembled using VelvetOptimiser v2.2 (24). The assembly statistics for all newly sequenced S. aureus genomes were shown in Table S1. CheckM v1.1.5 (25) accessed all genome data in our research. In this study, 328 published S. aureus genomes (China, *n* = 304; Asian cities, *n* = 23; America, *n* = 1) from NCBI database were collected. The criteria for genome selection were as follows: (i) genomes have full genome files of S. aureus, which were available in the NCBI database with GenBank annotation, and (ii) genomes of strains have information of isolation location. The 304 genomes of strains isolated from China were all eligible genome data that we could collect from NCBI. As a reference in epidemiological analysis, 24 genomes of strains isolated from foreign countries (96% from major Asian countries) were also collected.

### Phylogenetic analysis based on single-copy core gene families.

Orthologous groups were delimited using OrthoFinder v2.5.1 (25) with default parameters. The single-copy core gene families, core gene families, and pan-gene families were extracted based on the OrthoFinder output results. Nucleotide sequences of the single-copy core gene families were extracted and then aligned using MAFFT v7.475 (26). The phylogenetic analysis of S. aureus was performed using the single-nucleotide polymorphism (SNP) set present in 854 single-copy core gene families. The maximum likelihood (ML) tree was constructed using MEGA 7 software (27) (with the general time reversible [GTR] model).

### Core and pangenome analysis.

Heap’s law for pangenome models was used to analyze the pangenome (28). The total number of gene families (*n*, *y* axis) for increasing values of the number of genomes (*N*, *x* axis) is shown. The curve was a least-squares fit based on the power law (*n* = $\kappa N^{\gamma }$) to the averages. The core genome analysis was performed by regression analysis (29). A weighted least-squares regression by fitting the power law *n* = κ × exp(*m* × *N*) + Θ (*N*, the number of genomes; *n*, the number of core gene families; *Θ*, a constant value representing the predicted minimum number of core gene families; κ and *m*, parameters).

### Genetic population structure analysis and gene functional category.

The average nucleotide identity (ANI) was calculated using the JSpecies v1.2.1 software (30). Population structure analysis was conducted using the RhierBAPS v1.1.3 (31, 32). We analyzed the functional category of the gene family based on the cluster of orthologous groups (COG) assignment (33). The functional annotation of proteins was performed using an eggNOG-mapper v2.1.9 with default parameters (34, 35). The phage search tool enhanced release (PHASTER, http://phaster.ca/) was utilized to find the prophages (36). Genomic islands were predicted using the IslandViewer 4 (https://www.pathogenomics.sfu.ca/islandviewer/) database (37) using default parameters.

### Identification of virulence genes and resistance genes.

To identity the virulence genes and resistance genes, protein sequences of all Staphylococcus aureus genomes were aligned using BLASTp against the data set from the Virulence Factors Database (VFDB) (http://www.mgc.ac.cn/cgi-bin/VFs/v5/main.cgi) (38) and the Comprehensive Antibiotic Resistance Database (CARD) (https://card.mcmaster.ca/) (39) with the following three screening thresholds: (i) percentage of identical <50% or coverage <60%, (ii) percentage of identical >50% and percentage of identical <75% and coverage >60%, and (iii) percentage of identical >75% and coverage >60%. All blast results had E value cutoffs of <1e−6. These results were visualized using the R packages pheatmap v1.0.12 and Adobe Illustrator CS6.

### Comparative core genome analysis.

Comparative core genome analysis at branch points on the phylogenetic tree was performed to examine the differences in genetic characteristics among each subgroup. Increased and decreased core gene families at each branch point were extracted and annotated by the KEGG database (https://www.kegg.jp/); the following two different classification methods counted results: “gene function” and “pathway” and are shown in a bar chart. Based on the gene data above, relative enrichment ratio (RER) was introduced to describe the correlation between gene enrichment and pathway based on the following formula (shown in a line chart): RER = (number of genes annotated in this pathway) × 100/(total number of genes in this pathway); RER results were screened with 8 as the threshold (maximum number of peaks that can be retained), and the pathway corresponding to each peak was extracted. All figures were made by GraphPad Prism 9.0, R packages, and Adobe Illustrator CS6.

### Ethics approval and consent to participate.

The research protocol and informed consent were approved by the Ethics committee of the Tianjin Science and Technology Commission (approval number TMUaMEC2017017).

### Data availability.

All genome data of the 4 strains sequenced in this research have been uploaded to the NCBI database under project accession number PRJNA827471. GenBank assembly accession numbers of the data for the 4 genomes are as follows: Staphylococcus aureus ZF7406, GCA_023093755.1; Staphylococcus aureus B3-1, GCA_023093735.1; Staphylococcus aureus B3-1, GCA_023093705.1; and Staphylococcus aureus BD52508, GCA_023093785.1.
