# Supplementary material for: Molecular Epidemiology of Staphylococcus aureus in China Reveals the Key Gene Features Involved in Epidemic Transmission and Adaptive Evolution
Source: Microbiol Spectr. 2022 Oct 3;10(5):e01564-22. doi: 10.1128/spectrum.01564-22 (PMC9603185; doi:10.1128/spectrum.01564-22)
Supplement: Supplemental file 1 — Supplemental Material Figure. Download spectrum.01564-22-s0001.pdf, PDF file, 0.7 MB [file spectrum.01564-22-s0001.pdf]

SUPPLEMENTAL MATERIAL FIGURE

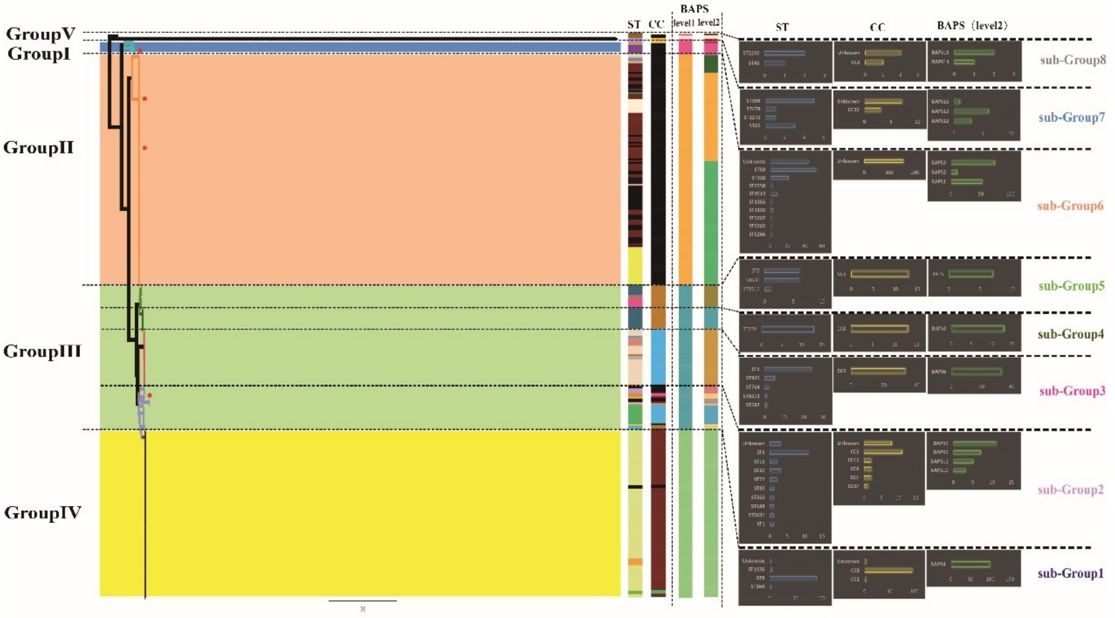

Figure S1 The information and statistics of MLST typing, CC typing and BAPS of 332 *Staphylococcus aureus* strains. Different Groups was labeled on left and different sub-Groups was labeled on right.

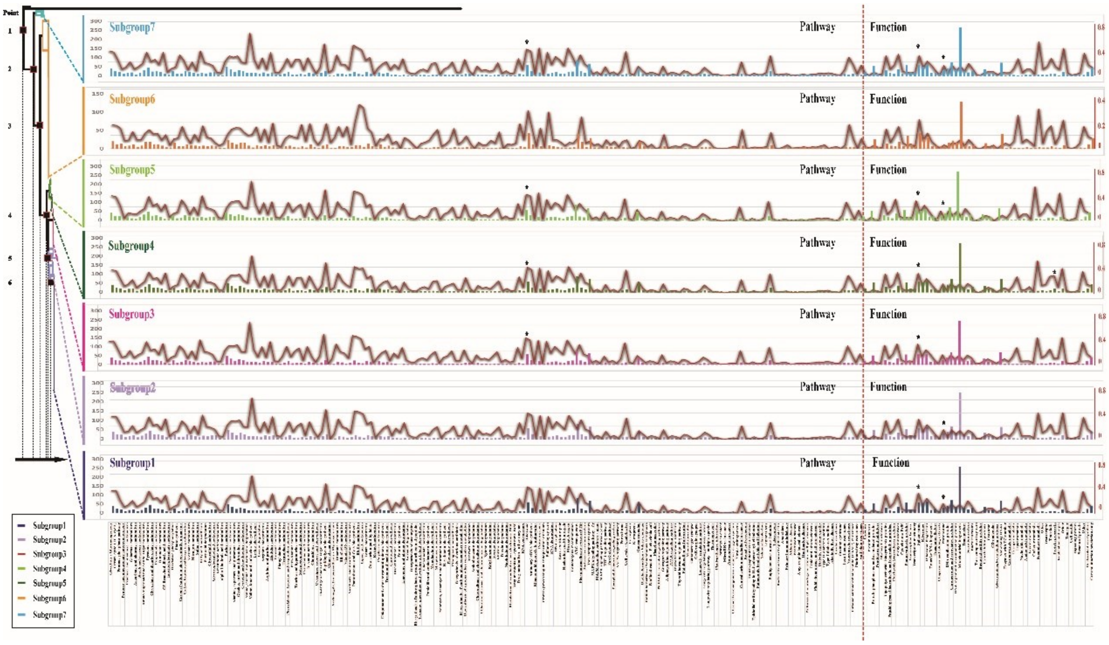

Figure S2 Core-genome analysis of each sub-Group based on KEGG annotation. The histogram represented the gene-number of each KEGG pathway and the color of each

sub-Group corresponded to the branch color on the tree. The line chart colored in darkred was represented the RER values of each KEGG pathway. *: Fisher's exact test P-value < 0.05, **: Fisher's exact test P-value < 0.01. Detailed information on the KEGG pathway was shown at the bottom.
